# Spectral Density Function Analysis Reveals Coupled Relaxation and Resonance Modes in Fluorinated Elastomers: Comparison With Semicrystalline Poly(tetrafluoroethylene)

**DOI:** 10.1002/mrc.70037

**Published:** 2025-09-09

**Authors:** Natsuki Kawabata, Naoki Asakawa, Teruo Kanki

**Affiliations:** ^1^ Materials Science Program, Graduate School of Science and Technology Gunma University Gunma Japan; ^2^ Institute of Scientific and Industrial Research Osaka University Osaka Japan

**Keywords:** ^19^F, depth profiling, fluoroelastomer, NMR, polymer films, PTFE, static magnetic field gradient

## Abstract

We reveal contrasting behaviors in molecular motion between the two materials, including the identification of resonance‐enhanced dynamic features in elastomers. We present a depth‐resolved analysis of molecular dynamics in semicrystalline polytetrafluoroethylene (PTFE) and fully amorphous fluorinated elastomer (SIFEL) films using static‐gradient solid‐state ^19^F NMR imaging. By measuring spin–lattice relaxation rates ( $R_{1}$) at multiple frequencies and evaluating the corresponding spectral density functions, we reveal distinct dynamic behaviors between the two materials. PTFE exhibits pronounced depth dependence in $R_{1}$, indicating enhanced molecular motion near the surface due to a structurally disordered amorphous layer. In contrast, the fluorinated elastomer shows spatially uniform $R_{1}$ values, reflecting its homogeneous molecular mobility. Notably, the elastomer's spectral density function contains resonance‐like peaks at finite frequencies, suggesting the presence of intrinsic vibrational modes superimposed on stochastic motion. This hybrid dynamic signature, captured through nuclear magnetic resonance (NMR) relaxation, offers a unique fingerprint of the elastomer's viscoelastic behavior. Our results demonstrate that static‐gradient NMR imaging can probe subtle spatial variations in polymer dynamics noninvasively and with high sensitivity, enabling direct comparison between crystalline and amorphous systems. The findings provide new insights into nanoscale surface dynamics and contribute to the development of advanced materials with tailored thermomechanical properties.

## Graphical abstract

We used static‐gradient ^19^F NMR imaging to compare molecular dynamics in semicrystalline polytetrafluoroethylene (PTFE) and amorphous fluorinated elastomer films. PTFE shows depth‐dependent motion, while the elastomer exhibits uniform dynamics with resonance‐like peaks in its spectral density, suggesting intrinsic vibrational modes.

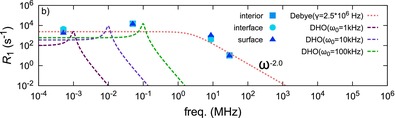

## 1 Introduction

The performance and functionality of polymeric materials largely depend on their molecular dynamics. In particular, phenomena such as glass transition, crystallization, and chain dynamics significantly influence the mechanical, thermal, optical, and electrical properties of these materials [1, 2]. Accurately understanding and controlling these molecular motions is essential for the design and optimization of new polymeric materials. However, elucidating such dynamics in detail is challenging due to their occurrence over a wide range of spatial and temporal scales, from the nanoscale to the microscale [3].

To address these challenges, solid‐state nuclear magnetic resonance (NMR) spectroscopy has emerged as a powerful analytical technique. Solid‐state NMR offers nondestructive, localized molecular‐level information and enables detailed analysis of molecular motion in both crystalline and amorphous polymers [4, 5]. In particular, the spectral density function, which reflects the characteristics of molecular motion, is a crucial parameter. By precisely analyzing this function, the dynamics of not only polymeric materials but also various other systems can be better understood [6, 7].

Traditional techniques such as spectroscopy and viscoelastic measurements have also been used to study molecular dynamics, but they generally provide macroscopic insights, limiting access to detailed molecular‐level information.

Among studies employing solid‐state NMR, the analysis of the spectral density function is especially important for understanding dynamic properties of materials. The spectral density function quantitatively describes the frequency dependence of molecular relaxation processes and internal motions, thereby elucidating the behavior of polymer chains and their interactions with the surrounding environment [8, 9].

In this study, we aim to analyze the molecular dynamics of a fully amorphous fluorinated elastomer using solid‐state NMR and to determine its spectral density function. Typically, molecular motion is observed as relaxation, leading to spectral density functions with maxima at zero frequency. However, in this study, we found that the spectral density function derived from spin–lattice relaxation includes resonance‐like peaks superimposed due to intrinsic vibrations of the elastomer.

**FIGURE 1:**
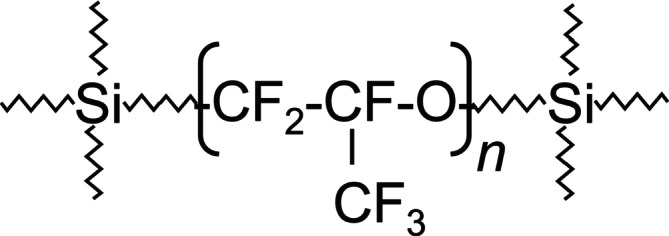
Chemical structure of SHIN‐ETSU SIFEL.

Additionally, our previous studies on polytetrafluoroethylene (PTFE) films revealed that rotational motion is more excited near both the air‐side surface and the substrate interface compared to the film interior. While no significant differences in rotational dynamics were found between the two interfaces, translational diffusion behavior differed [10]. In contrast, this study attempts to image the depth dependence of the spectral density function for the molecular dynamics of a fluorinated elastomer. Our findings indicate that, unlike PTFE, the rotational dynamics of the SHIN‐ETSU SIFEL (see Figure 1 for chemical structure) film do not differ significantly between the surface and the interior.

## 2 Experimental

### 2.1 Principle of Depth‐Resolved Imaging

Recent developments in compact NMR spectrometers have been remarkable [11], and the specialized spectrometer used in this study is likely part of that trend. In addition, there have already been studies that have attempted depth profiling using well logging [12]. Our study performs multifrequency NMR relaxation measurements using a permanent magnet as a magnetic field gradient source within an electromagnet and is positioned to establish a methodology known as spectral density function imaging.

**FIGURE 2:**
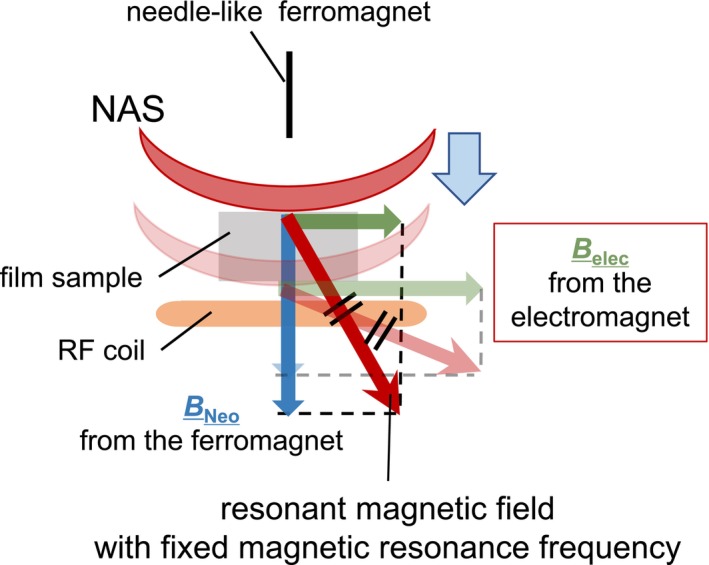
The combined effect of the magnetic field generated by the needle‐like ferromagnet, $B_{\mathrm{Neo}}$, and the static magnetic field from the electromagnet, $B_{\text{elec}}$ gives rise to the resonant field, NAS. The NAS morphology, shaped by the needle‐like ferromagnet, exhibits a meniscus‐lens‐like structure. By adjusting the strength of the static magnetic field from the electromagnet, the NAS can be vertically displaced relative to the sample plane. When the static magnetic field from the electromagnet is weak, the magnetic field generated by the needle‐like ferromagnet dominates, causing the NAS to shift upward, closer to the needle. Conversely, when the static magnetic field is strong, a weaker contribution from the needle‐like ferromagnet suffices, leading to a downward displacement of the NAS, away from the needle.

The sample experiences a magnetic field gradient resulting from the combined effects of a static external field and a gradient induced by a needle‐shaped ferromagnet. This setup creates a specific region, the NMR active slice (NAS), where the field strength is sufficient for NMR detection (Figure 2). The NAS, with a concave shape influenced by the ferromagnet, allows characterization of spin density or molecular dynamics at specific depths. By adjusting the external magnetic field, the NAS can be vertically shifted, enabling depth‐resolved NMR analysis of the sample.

### 2.2 Experimental Setup

The experimental setup is shown in Figure 2. A spherical neodymium magnet (8‐mm diameter, from TRUSCO Nakayama) and an iron needle (1‐mm diameter, tip 0.2 mm) were mounted on an aluminum jig. When brought into contact, the needle becomes magnetized and acts as a localized ferromagnetic source. This needle was placed between the poles of a water‐cooled electromagnet, which provided both the static magnetic field and its gradient. Detailed apparatus specifications are available in our previous publications [10, 13].

### 2.3 MRI Measurements

We first calibrated the resonance frequency to 29.65 MHz and measured the spin–lattice relaxation rate ( $R_{1}$) using the multiple‐pulse saturation recovery method. The external static magnetic field was incrementally increased via a water‐cooled electromagnet, with signal integration performed 256 times per point.

Measurements were also conducted at 8.7065 MHz using the same pulse sequence and parameters as for the 29.65‐MHz experiments.

Next, we used the spin‐lock method under similar conditions to determine the spin–lattice relaxation rate $R_{1\rho }$ in the rotating frame [14], with a spin‐locking frequency of ∼ 50 kHz and a resonance frequency of 29.65 MHz.

Lastly, the Jeener–Broekaert method was applied under the same conditions to determine the spin–lattice relaxation rate $R_{1D}$ for the ^19^F–^19^F dipolar order [15]. The pulse sequence was π/2 ‐ $\tau_{1}$ ‐ π/4 ‐ $\tau_{2}$ ‐ π/4 ‐ $\tau_{1}$‐ (acquisition), with $\tau_{1}$ set to 30 μs and $\tau_{2}$ acting as a recovery time. The ^19^F–^19^F dipolar interaction frequency in the PTFE film was 0.514 kHz. The setting for $\tau_{1}$ was determined based on the $R_{2}$ value of PTFE. The $R_{2}$ value exhibits τ dependence due to the diffusion behavior of the amorphous regions of PTFE, yielding $R_{2}\approx 10^{3}‐10^{4}\;s^{-1}$, or equivalently $T_{2}=100\;\mu s$‐1 ms; these data will be published elsewhere. A ccordingly, we set $\tau_{1}=30\;\mu s.$ Although $R_{2}$ has not yet been measured for SIFEL, its NMR linewidth is significantly narrower than that of PTFE, as expected for an elastomer. Therefore, we anticipate that $T_{2}$ is larger than in PTFE, supporting the appropriateness of using $\tau_{1}=30\mu s$ in our experiments.

## 3 Results and Discussion

### 3.1 Molecular Dynamics and Structural Homogeneity of Polymeric Materials Based on Depth‐Dependent $R_{1}$ Relaxation Rates

**FIGURE 3:**
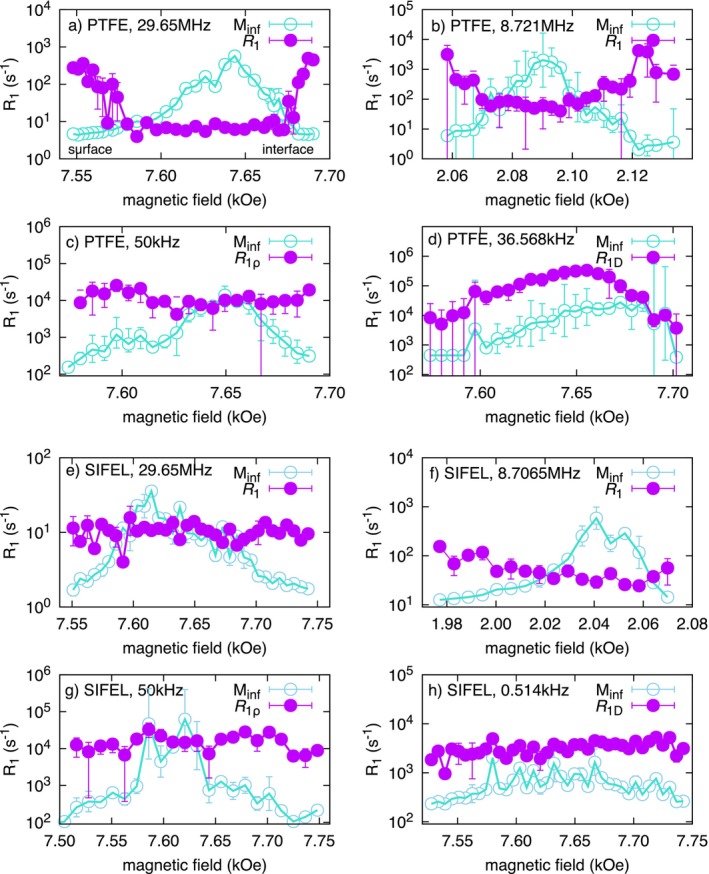
Results of variable frequency $R_{1}$ measurements. Comparison of $R_{1}$ between PTFE and SIFEL. Magnetization intensity at arbitrary unit, $M_{\inf}$, is also shown. Panels b and d of PTFE were reproduced from Figure 4b,d in Ref. [10] with permission.

In this study, we observed the depth‐dependent variation in $R_{1}$ relaxation rates for a crystalline PTFE film and fully amorphous fluorinated elastomer film, both with a thickness of 2 mm, and examined the molecular dynamics and structural characteristics of each material. As shown in Figure 3, the depth dependence of $R_{1}$ relaxation rates revealed notable differences between the two materials under investigation.

Figure 3a–d showed that the $R_{1}$ values of PTFE films were shorter near the surface and decreased progressively toward the interior [10]. In contrast, the elastomer exhibited no clear depth‐dependent trend within the measured range, indicating spatially uniform relaxation behavior.

Figure 3a,b show that, at resonance frequencies above 8.7 MHz, the $R_{1}$ values of PTFE films are larger near the surface and gradually decrease toward the interior [10]. At 50 kHz, however, the $R_{1\rho }$ values become insensitive to film depth and even exhibit the opposite trend for $R_{1D}$ at 36.568 kHz. These behaviors arise from differences in the power spectral density of molecular fluctuations between the surface and interior regions of the film.

#### 3.1.1 Surface Effects in Bulk Samples

Generally, in glassy polymer thin films, it is known that as film thickness decreases, the glass transition temperature ( $T_{g}$) also decreases, and the mobility of surface molecules becomes markedly different from that of the bulk [16, 17, 18, 19, 20]. Conversely, at the substrate interface, polymer chains experience orientational constraints due to interfacial interactions, leading to an increase in $T_{g}$ [21, 22, 23, 24]. These behaviors reflect enhanced segmental dynamics near the free surface and suppressed mobility near the substrate interface.

Furthermore, as film thickness increases, surface effects often diminish, and the overall dynamics approach those of bulk polymers. However, this apparent attenuation of surface effects in thicker films raises an important question: Is the reduction of surface dynamics a genuine physical phenomenon, or is it merely a reflection of the increasing difficulty in experimentally isolating surface‐specific signals due to the dominance of bulk contributions?

This issue has been addressed in several studies using high spatial resolution and high‐sensitivity techniques. For example, Ellison and Torkelson [25] demonstrated the distribution of $T_{g}$ values in confined glass‐forming materials at the nanoscale, suggesting that surface‐induced dynamic heterogeneity persists even in relatively thick films. Similarly, Fakhraai and Forrest [26] revealed the enhanced surface molecular mobility using advanced experimental methods, supporting the notion that surface effects are not merely due to instrumental limitations. These findings suggest that, while experimental challenges certainly exist, the reduction in surface effects with increasing thickness likely reflects a genuine physical crossover from surface‐dominated to bulk‐dominated behavior.

Recent studies have shown that the free surface of polymers exhibits enhanced molecular mobility even in films much thicker than the typical nanoscale range. While earlier research focused on ultrathin films with high surface‐to‐volume ratios, recent evidence suggests that enhanced molecular mobility at the free surface persists even in films with micrometer‐scale thicknesses. For instance, Chai et al. demonstrated that nanometer‐thick surface layers of polystyrene maintain flowability at temperatures much lower than the bulk $T_{g}$, even in films several hundred nanometers thick, by directly measuring surface flow dynamics using a stepped film structure [27]. Similarly, Yang et al. showed that the observed reduction in $T_{g}$ in unentangled polystyrene films results from enhanced segmental dynamics at the free surface, which remains influential despite increasing film thickness [28].

These findings strongly support the notion that surface effects do not necessarily vanish in thick films but instead persist as genuine physical properties that can influence both local and overall polymer dynamics.

### 3.2 Structural Inhomogeneity and Relaxation Time Variation in PTFE Films

PTFE is a material with high crystallinity and rigid polymer chains, and its crystalline structure tends to strongly restrict molecular motion. The low $R_{1}$ values observed in the interior likely reflect this constrained mobility resulting from a high degree of crystallization.

In contrast, the $R_{1}$ values near the surface are higher, indicating relatively enhanced molecular mobility. This phenomenon can be explained by the presence of a “surface amorphous layer” typically observed in crystalline polymers [29, 30, 31, 32]. Crystalline structures are less likely to form at the surface, leading to an increased fraction of amorphous components, which in turn promotes greater molecular freedom and faster relaxation. Therefore, the depth‐dependent $R_{1}$ behavior in PTFE reflects structural and dynamic anisotropy stemming from inhomogeneous crystalline order between the surface and interior.

The surface structure of highly crystalline polymers such as PTFE has long attracted attention due to its unique combination of properties, including exceptional chemical inertness, low surface energy, and high wear resistance. Although PTFE is known for its crystallinity exceeding 90%, several studies suggest that its surface may contain disordered or amorphous‐like layers that differ from the highly ordered bulk phase [29, 30, 31, 32].

Such a surface amorphous layer is believed to arise from disrupted crystalline order due to imperfect packing of polymer chains near the surface. Korolkov, using atomic force microscopy, analyzed the surface morphology of PTFE films and identified low‐density, structurally irregular regions suggestive of amorphous domains [29]. Although molecular mobility in this layer is still constrained by the inherent rigidity and helical structure of PTFE chains, its existence may play a significant role in surface phenomena such as adhesion, tribological behavior, and surface modification via plasma or UV treatment. Furthermore, the presence of this layer provides a plausible explanation for the relatively high responsiveness of the PTFE surface to physical and chemical surface treatments, despite its inherent inertness.

These findings suggest that even in polymers exhibiting extremely high crystallinity, the outermost surface can significantly deviate from the bulk structure and form a thin but functionally important amorphous‐like layer.

PTFE is also known to possess a surface amorphous layer often referred to as a “weak boundary layer” (WBL), which has been reported to significantly affect its adhesion properties and surface modification behavior. Ohkubo et al. demonstrated that plasma treatment in combination with heating dramatically improves the adhesion between PTFE and isobutylene‐isoprene rubber by removing the WBL and hardening the PTFE surface [32]. Similarly, Hubert et al. showed that the amorphous components of the PTFE surface are preferentially etched during plasma treatment, indicating their higher reactivity compared to crystalline regions [30]. Additionally, Grytsenko et al. reported that vapor‐deposited PTFE films exhibit enhanced amorphous behavior and diverse structural characteristics under specific conditions [31]. These studies provide strong evidence for the presence of structurally distinct amorphous surface layers in PTFE and offer important insights into the interfacial properties and behaviors during surface treatment.

**FIGURE 4:**
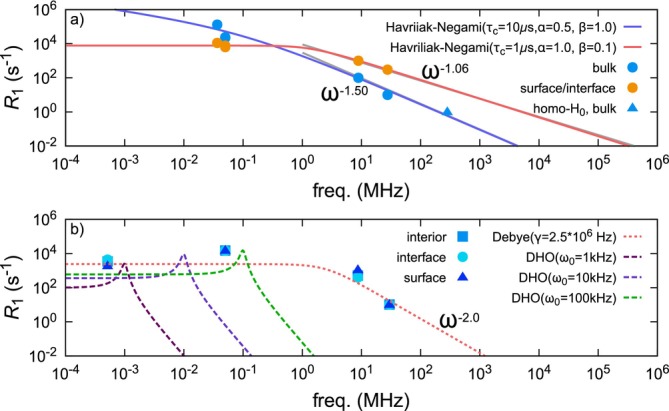
Spectral density function obtained from variable‐frequency spin–lattice relaxation measurements for PTFE (a) and SIFEL (b). No depth‐dependece was observed on the power spectral density for SIFEL while distinctive power spectral densities between interior and surface/interface for PTFE film were observed. For SIFEL, at high frequencies above approximately 8.7 MHz, the spectrum corresponds to a single Debye‐type relaxation mode with a characteristic correlation time. At low frequencies, resonance‐like peaks are superimposed. As a guide, resonance spectra based on a damped harmonic oscillator (DHO) model with several intrinsic frequencies are shown. The parameters used for the resonant spectra from low to high frequency are as follows: ( $\omega_{0},\gamma ,A_{\text{reson}}$) = (1 kHz, 100 Hz, $5\times 10^{-7}$), (10 kHz, 1 kHz, $1.8\times 10^{-3}$), and (100 kHz, 10 kHz, $3.0\times 10^{0}$). These spectra are interpreted as the sum of resonant contributions and a single relaxation spectrum. Panel (a) was reproduced basd on Figure 5 in Ref. [10] with permission.

Taken together, these findings clarify that molecular dynamics near the air‐side surface and substrate interface are more excited than in the film interior, with rotational motion contributing to the increase in $R_{1}$. Indeed, our previous study concerning variable frequency $R_{1}$ measurements for the PTFE film indicates that the spectral density function for the surface/interface is distinctive to that of the interior of the film (Figure 4a). As shown in Figure 4a, the power‐law behavior of PTFE is likely related to the distribution of correlation times of molecular motion. Since the exponent is smaller at the surface/interface (−1.06) than in the interior (−1.50), we infer that the distribution of correlation times is broader near the surface/interface, indicating greater heterogeneity in molecular dynamics.

However, the thickness of the surface and interface layers observed via NMR reaches several hundred micrometers, which differs significantly from the actual nanometer‐scale thicknesses observed by other techniques. This discrepancy arises from the strong influence of ^19^F–^19^F spin diffusion on $R_{1}$ relaxation in NMR, and care must be taken when interpreting the results. Nevertheless, the fact that NMR can access information from the nanometer‐scale surface region without using nanoscale measurement techniques is particularly intriguing.

### 3.3 Molecular Structure and Relaxation Behavior of Elastomers

Figure 3e–h shows the depth‐resolved $R_{1}$ imaging results for the thermally cured fluorinated elastomer SIFEL (#2617) at 423 K for 1 h. The radius and thickness of the SIFEL sample with disk‐like shape were 15.0 and 2.0 mm. In contrast to PTFE, no inhomogeneity in molecular motion was observed along the depth direction. Elastomers are polymeric materials characterized by flexible polymer chains and crosslinked structures, which result in relatively uniform spatial molecular mobility. The lack of depth dependence in $R_{1}$ relaxation rates suggests that the distribution of free volume and local relaxation modes is homogeneous throughout both the surface and interior of the material.

In addition, elastomers are known for properties such as self‐healing ability and flexible responses to external stress, which may also contribute to the uniformity of molecular dynamics at the molecular level. Thus, in the case of SIFEL, the primary physical factors governing molecular motion appear to be spatially uniform throughout the material, and the absence of depth dependence in $R_{1}$ aligns with the structural and mechanical homogeneity of the system.

Another noteworthy observation pertains to the spectral density function derived from the experimental data. In many cases, molecular motion is observed as a relaxation process, yielding a spectral density function with a peak at zero frequency. However, the spectral density function obtained in this study exhibits peaks at finite frequencies (Figure 4b).

This finding indicates that the molecular motions affecting ^19^F NMR relaxation are not entirely random but include vibrational components coupled with stochastic motion. A plausible explanation is that the resonances originate from intrinsic vibrational modes of the elastomer. In other words, resonance‐like peaks due to intrinsic vibrations of the elastomer are superimposed on the spectral density function governing spin fluctuations.

Specifically, at high frequencies above approximately 8.7 MHz, the function exhibits an $\omega^{-2}$ dependence, characteristic of a single Debye‐type relaxation mode with a unique correlation time $\tau_{c}$ ( $=\gamma^{-1}$, where γ corresponds to the damping coefficient) [see Equation 2]. On the low‐frequency side, however, the spectrum includes resonance peaks (see Equation (3)). For a guide for eyes, the figure includes resonance spectra modeled using forced damped harmonic oscillators (DHO) with several characteristic frequencies.

The overall spectral density function $J\left(\omega \right)$ can be interpreted as a sum of multiple resonance spectra $J_{\text{reson}}\left(\omega ,\omega_{0}\right)$, each corresponding to a particular resonant frequency $\omega_{0}$ and damping constant γ and a single relaxation spectrum $J_{\text{relax}}\left(\omega \right)$ characterized by a single correlation time:

(1) $$J\left(\omega \right)=J_{\text{relax}}\left(\omega \right)+\sum_{\omega_{0}}\;J_{\text{reson}}\left(\omega ,\omega_{0}\right)$$

with

(2) $$J_{\text{relax}}\left(\omega \right)=A_{\text{relax}}\cdot \frac{1}{\gamma^{2}+\omega^{2}}$$

(3) $$J_{\text{reson}}\left(\omega ,\omega_{0}\right)=A_{\text{reson}}\cdot \frac{\gamma }{{\left(\omega_{0}^{2}-\omega^{2}\right)}^{2}+{\left(\gamma \omega \right)}^{2}}$$

(4) $$\gamma =\tau_{c}^{-1}.$$

In contrast to PTFE, because SIFEL is fully amorphous, both the correlation function distribution parameters and the spectra of resonant motions specific to elastomers are superimposed on the correlation function. Consequently, the full correlation function cannot be determined due to its complexity, and fitting curves are not presented. Nevertheless, the observed decrease in $R_{1}$ at low frequencies strongly suggests that resonant motion contributes to spin–lattice relaxation.

This spectral structure represents the extent to which the vibrational energy of the elastomer dissipates thermally via spin relaxation. Therefore, it may serve as a microscopic indicator for evaluating macroscopic thermomechanical properties of elastomers.

## 4 Conclusion

In this study, the molecular dynamics of fluorinated elastomer films were investigated using NMR imaging under a static magnetic field gradient. Depth‐dependent longitudinal relaxation rates ( $R_{1}$) were analyzed and compared with those of PTFE films. As a result, significant differences were revealed between the two materials. PTFE exhibited depth‐dependent relaxation behavior, indicating structural and dynamic inhomogeneity particularly accelerated molecular motion near the surface due to the presence of an amorphous layer. In contrast, the fluorinated elastomer (SIFEL) showed uniform $R_{1}$ values throughout the entire film thickness, suggesting spatially homogeneous molecular mobility likely resulting from its flexible backbone and crosslinked network.

Moreover, the spectral density function derived from the NMR measurements revealed not only relaxation‐dominated behavior in the elastomer but also resonance peaks at finite frequencies. This indicates that the molecular motion within the fluorinated elastomer includes vibrational modes, likely reflecting the material's intrinsic mechanical resonances. Such coupling between vibrational modes and molecular motion may influence energy dissipation mechanisms and could serve as a novel indicator of thermomechanical properties.

Our findings demonstrate that solid‐state NMR imaging provides powerful insights into the spatial variation of polymer dynamics and can detect subtle differences between crystalline and amorphous structures. The absence of depth‐dependent molecular mobility in fluorinated elastomers stands in contrast to the behavior observed in highly crystalline PTFE, highlighting the influence of polymer structure on local dynamics. Furthermore, the identification of resonance‐enhanced relaxation peaks opens new avenues for evaluating polymer functionality via dynamic molecular signatures.

These results contribute to a deeper understanding of both surface and bulk dynamics in polymer films and underscore the utility of NMR‐based spectral density analysis for advanced material characterization.

## Conflicts of Interest

The authors declare no conflicts of interest.

## Peer Review

The peer review history for this article is available at https://www.webofscience.com/api/gateway/wos/peer‐review/10.1002/mrc.70037.

## Data Availability

The datasets generated and analyzed during the current study are available from the corresponding author on reasonable request.
